# A *Very Oil Yellow1* Modifier of the *Oil Yellow1-N1989* Allele Uncovers a Cryptic Phenotypic Impact of *Cis*-regulatory Variation in Maize

**DOI:** 10.1534/g3.118.200798

**Published:** 2018-12-05

**Authors:** Rajdeep S. Khangura, Sandeep Marla, Bala P. Venkata, Nicholas J. Heller, Gurmukh S. Johal, Brian P. Dilkes

**Affiliations:** * Department of Botany and Plant Pathology, Purdue University, IN 47907; † Center for Plant Biology, Purdue University, IN 47907; ‡ Department of Biochemistry, Purdue University, IN 47907

**Keywords:** Chlorophyll biosynthesis, cryptic variation, cis-acting, complex traits, epistasis

## Abstract

Forward genetics determines the function of genes underlying trait variation by identifying the change in DNA responsible for changes in phenotype. Detecting phenotypically-relevant variation outside protein coding sequences and distinguishing this from neutral variants is not trivial; partly because the mechanisms by which DNA polymorphisms in the intergenic regions affect gene regulation are poorly understood. Here we utilized a dominant genetic reporter to investigate the effect of cis and *trans*-acting regulatory variation. We performed a forward genetic screen for natural variation that suppressed or enhanced the semi-dominant mutant allele *Oy1-N1989*, encoding the magnesium chelatase subunit I of maize. This mutant permits rapid phenotyping of leaf color as a reporter for chlorophyll accumulation, and mapping of natural variation in maize affecting chlorophyll metabolism. We identified a single modifier locus segregating between B73 and Mo17 that was linked to the reporter gene itself, which we call *very oil yellow1* (*vey1*). Based on the variation in OY1 transcript abundance and genome-wide association data, *vey1* is predicted to consist of multiple *cis*-acting regulatory sequence polymorphisms encoded at the wild-type *oy1* alleles. The *vey1* locus appears to be a common polymorphism in the maize germplasm that alters the expression level of a key gene in chlorophyll biosynthesis. These *vey1* alleles have no discernable impact on leaf chlorophyll in the absence of the *Oy1-N1989* reporter. Thus, the use of a mutant as a reporter for magnesium chelatase activity resulted in the detection of expression-level polymorphisms not readily visible in the laboratory.

Gene function discovery via mutant analyses focuses on linking phenotype alterations to gene variants. Forward genetics has been of great value to understand biological systems but is predominantly useful for determining functions for genes with alleles that cause large phenotypic impacts. Natural variants, including those that encode alleles of relevance to adaptation and fitness of the organism, are often of small effect (Fisher 1930; Orr 1998, 2005). Mutant alleles with conditional impacts on phenotypes through genetic interactions or modifiers, as well as alleles of small individual effect are difficult to study. Further, when studies of natural variation identify loci that have not been previously associated with a biological process, it can be difficult to definitively associate such genetic variants with physiological and biochemical mechanisms.

A forward genetics approach that uses a mutant phenotype as a reporter for genetic interactions can be used to detect modifiers in natural populations and expose cryptic variation affecting traits of interest (Johal *et al.* 2008). This Mutant-Assisted Gene Identification and Characterization (MAGIC) approach is particularly efficient in species where outcrossing is easy, such as maize (*Zea mays*). Although this approach can be applied to any mutant with a quantifiable phenotype, it is convenient when a dominant mutant allele is used as a reporter because natural variants that encode enhancers or suppressors of a given mutant phenotype can be detected in F1 crosses. Any germplasm collection, diversity panel, or line-cross population can serve as the variable parents in these crosses to provide segregating alleles for mapping loci that alter mutant phenotype expression. Natural variants discovered by this type of genetic screen have an experimental link (*e.g.*, genetic modifiers) of the processes affected by the mutant reporter allele. Thus, this approach speeds the assignment of a mechanism to natural variation in the germplasm. Indeed, one can consider this as a screen for epistatic or contingent gene action. MAGIC screens have identified loci from maize involved in the hypersensitive response (Penning *et al.* 2004; Chintamanani *et al.* 2010; Olukolu *et al.* 2013, 2014, 2016) and plant development (Buescher *et al.* 2014), among others. In these cases, in the absence of a mutant allele, no phenotype was previously associated to the modifying alleles demonstrating the remarkable efficiency of this genetic screen to detect epistatic interactions Thus, this approach is a powerful way to both characterize cryptic variation in genomes and construct genetic pathways affecting phenotypes of interest.

The easy visual scoring and simplicity of quantifying chlorophyll make chlorophyll biosynthetic mutants an excellent reporter for MAGIC screens. Chlorophyll is a major component of central metabolism in plants, which can produce phototoxic intermediates during both synthesis (Hu *et al.* 1998; Huang *et al.* 2009) and breakdown (Gray *et al.* 1997; Mach *et al.* 2001; Gray *et al.* 2002; Yang *et al.* 2004; Sindhu *et al.* 2018), and its levels are carefully regulated in plants (Meskauskiene *et al.* 2001). Enzymatic conversion of protoporphyrin IX into magnesium protoporphyrin IX by Magnesium Chelatase (MgChl) is the first committed step in chlorophyll biosynthesis (Von Wettstein *et al.* 1995). MgChl is a hetero-oligomeric enzyme consisting of three subunits (I, D, and H) that are conserved from prokaryotes to plants. The MgChl subunit I is encoded by the *oil yellow1* (*oy1*; GRMZM2G419806) gene in maize and encodes the AAA+-type ATPase subunit that energizes the protein complex (Fodje *et al.* 2001). Weak loss-of-function alleles of *oy1* result in recessive yellow-green plants while complete loss-of-function alleles result in a recessive yellow seedling-lethal phenotype (Sawers *et al.* 2006). The semi-dominant *Oy1-N1989* allele carries a leucine (L) to phenylalanine (F) change at amino acid position 176 (L176F). Heterozygous plants carrying one *Oy1-N1989* allele and one wild-type allele are oil-yellow, but *Oy1-N1989* homozygotes lack any MgChl activity and die as yellow seedlings with no chlorophyll accumulation (Sawers *et al.* 2006). The orthologous L->F mutation (encoded by *Oy1-N1989*) was identified in barley (L161F) and also results in a pale-green phenotype as a heterozygote and yellow seedling-lethal phenotype as a homozygote with no detectable chlorophyll (Hansson *et al.* 1999). The biochemical basis of this semi-dominant mutant allele was studied by creating a mutant MgChl subunit I in *Rhodobacter* (BCHI), with the orthologous amino acid change at position 111 of wild-type BCHI (Hansson *et al.* 2002). The L111F mutation converted BCHI into a competitive inhibitor of MgChl that reduced enzyme activity by fourfold when mixed 1:1 with wild-type BCHI (Hansson *et al.* 2002; Lundqvist *et al.* 2013). This conserved leucine residue is between the ATP-binding fold created by the Walker A and B motifs of MgChl subunit I (Hansson *et al.* 2002; Sawers *et al.* 2006; Lundqvist *et al.* 2013), and its substitution with phenylalanine was deleterious to dephosphorylation activity. The ATPase activity of wild-type MgChl complex is directly proportional to the magnesium chelation reaction. However, complexes assembled from MgChl subunit I with the L->F change exhibit reduced ATPase activity and no ability to chelate Mg${}^{2+}$ ions into protoporphyrin IX (Hansson *et al.* 1999, 2002; Sawers *et al.* 2006). The absence of MgChl activity displayed by the mutant BCHI carrying L111F substitution (BCHI${}^{L111F}$) demonstrates that this amino acid change results in a dominant-negative subunit which uncoupled ATP hydrolysis and magnesium chelation (Hansson *et al.* 2002; Lundqvist *et al.* 2013).

We screened maize germplasm for cryptic variation using the *Oy1-N1989* mutant as a dominant reporter for chlorophyll biosynthesis. We hypothesized that alteration in the quantity of biochemically active MgChl complex should change chlorophyll content and plant color in heterozygous *Oy1-N1989* mutants. For instance, in a population of heterozygous *Oy1-N1989/oy1* F1 plants, an amino acid change in the wild-type OY1 protein that alters the dissociation constant (kD) of OY1 for the other protein subunits of MgChl should contribute to variance in chlorophyll biogenesis. Similarly, a *cis*-acting expression QTL (eQTL) that increases expression of the wild-type *oy1* allele should result in assembly of more active MgChl complexes and increase chlorophyll content in F1 mutant plants. Thus, chlorophyll content of *Oy1-N1989* mutants should be modulated by the stoichiometry of the wild-type and mutant OY1 proteins present in the MgChl complex in heterozygous *Oy1-N1989/oy1* plants affected either by protein structure variation or abundance changes.

We introgressed the maize *Oy1-N1989* mutant allele into the B73 inbred background and maintained it as a heterozygote (*Oy1-N1989/oy1*:B73). While crossing this mutant to multiple backgrounds, we detected genetic variation in the *Oy1-N1989/oy1* mutant phenotype expression between the maize inbred lines B73 and Mo17. The phenotype of the *Oy1-N1989* mutant heterozygotes was suppressed in the B73 background. However, F1 hybrids with Mo17 dramatically enhanced it. We carried out genetic mapping experiments using five F1 populations. In each of these mapping experiments, we identified a quantitative trait locus (QTL) of large effect on chromosome 10 linked to the *oy1* locus itself. The inheritance of the traits, proposed allele expression bias at *oy1* due to a putative *cis*-acting regulatory element, implications of the discovered cryptic variation, and the utility of this study, in general, are discussed.

## Materials and Methods

### Plant Materials

The *Oy1-N1989* mutant allele was acquired from the Maize Genetics Cooperation Stock Center (University of Illinois, Urbana-Champaign, IL). The original allele of the *Oy1-N1989* mutation was isolated from a *r1 c1* colorless synthetic stock of mixed parentage (Myron Gerald Neuffer, personal communication). This allele was introgressed into B73 for eight generations by repeated backcrossing of B73 ear-parents with *Oy1-N1989/oy1* pollen-parents and is maintained as a heterozygote (*Oy1-N1989/oy1*:B73) by crossing to wild-type siblings.

Line-cross QTL mapping: For these experiments, *Oy1-N1989/oy1*:B73 plants were crossed as pollen-parents to 216 Intermated B73 x Mo17 recombinant inbred lines (IBM) (Lee *et al.* 2002) and 251 Syn10 doubled haploid lines (Syn10) (Hussain *et al.* 2007) to generate F1 progenies. The QTL validation was done using F1 progenies derived from the cross of 35 B73-Mo17 Near-Isogenic lines (BM-NILs) as ear-parents crossed with pollen from *Oy1-N1989/oy1*:B73 plants. These BM-NILs consisted of 22 B73-like NILs and 13 Mo17-like NILs with introgression of the reciprocal parental genome (B73 or Mo17) and were developed by three repeated backcrosses into recurrent parent followed by four to six generations of self-pollination (Eichten *et al.* 2011).

Genome-wide association (GWA) mapping: For this experiment, *Oy1-N1989/oy1*:B73 plants were crossed to 343 inbred lines that included 249 inbreds from maize association panel (Flint-Garcia *et al.* 2005), and 94 inbred lines that included 82 Expired Plant Variety Protections (ExPVP) lines from the Ames panel (Romay *et al.* 2013). Pollen from *Oy1-N1989/oy1*:B73 plants were used for these crosses except for the popcorn lines in the maize association panel, where *Oy1-N1989/oy1*:B73 plants were used as an ear-parent to avoid the crossing barrier due to gametophytic factor GA1-S (first described by Correns in 1902) (Mangelsdorf and Jones 1926; Lauter *et al.* 2017). This panel of 343 inbred lines is referred to as maize diversity lines (MDL). The full list of IBM, Syn10, BM-NILs, and MDL used to develop F1 hybrid populations are provided in Tables S1-S4.

### Field Trials

All field experiments were performed at the Purdue Agronomy Center for Research and Education (ACRE) in West Lafayette, Indiana. All F1 populations described below were planted as a single plot of 12-16 plants that segregated for both mutant and wild-type siblings. Plots were sown in a 3.84 m long row with the inter-row spacing of 0.79 meters and an alley space of 0.79 meters. No irrigation was applied during the entire crop season as rainfalls were uniformly distributed for satisfactory plant growth. Conventional fertilizer, pest and weed control practices for growing field maize in Indiana were followed. Progenies of *Oy1-N1989/oy1*:B73 pollen-parents crossed with B73 and Mo17 were planted as parental checks in each block of every experiment. The testcross F1 populations with IBM were evaluated in a single replication in the summer of 2013 with each range treated as a block. In 2016, the testcross F1 populations with Syn10 lines were evaluated as two replications in a randomized complete block design (RCBD) with each range divided into two blocks. The testcross F1 progenies with BM-NILs and parents (B73 and Mo17) were planted in a RCBD with five replications in 2016. In the same year, F1 populations with MDL were also evaluated with three replications planted in a RCBD. Each replication of MDL F1 population was divided into ten blocks of the same size, and parental checks were randomized within each block.

### Phenotyping and Data Collection

Maize seedlings used for destructive and non-destructive chlorophyll quantification were grown under greenhouse conditions using mogul base high-pressure sodium lamps (1000 Watts) as the supplemental light source for L:D cycle of 16:8 and temperature around 28° (day-time) and 20° (night-time). Destructive chlorophyll measurements were performed on the fresh weight basis in 80% acetone solution using a UV-VIS spectroscopic method (Lichtenthaler and Buschmann 2001). Non-destructive chlorophyll measurements were performed in the middle of the same leaf avoiding the midribs using the procedure described below.

For the field-grown experiments, mutant siblings in the suppressing genetic backgrounds were tagged at knee height stage with plastic tags so that they can be easily distinguished from the wild-type siblings at later developmental stages. All the F1 families segregated for the mutant (*Oy1-N1989/oy1*) and wild-type (*oy1/oy1*) siblings in approximately 1:1 fashion. For each F1 family, two to four plants of each phenotypic class were picked at random for trait measurements. Non-destructive chlorophyll content in the maize leaves was approximated using a chlorophyll content meter model CCM-200 plus (Opti-Sciences, Inc., Hudson, NH). The measurements were expressed as chlorophyll content index (CCM). Measurements were taken on the leaf lamina of the top fully expanded leaf at two time points. First CCM measurements were taken at 25-30 days after sowing (expressed as CCMI) and the second at 45-50 days after sowing (expressed as CCMII). For each trait, measurements were performed on both mutant (reported with a prefix MT) and wild-type (reported with a prefix WT) siblings. Besides using primary trait measurements of CCMI and CCMII on mutant and wild-type siblings, indirect CCM measurements were also calculated and expressed as ratios (MT/WT) and differences (WT-MT) of CCMI and CCMII. Phenotypic data of all the CCM traits in the F1 populations with both bi-parental populations, BM-NILs and MDL are provided in Tables S1-S4.

### Public Genotypic and Gene Expression Datasets

Public marker data for the IBM was obtained from www.maizegdb.org (Sen *et al.* 2010). A total of 2,178 retrieved markers were reduced to 2,156 after the removal duplicates. Approximately 13.3% of the marker data were missing. The reads per kilobase of transcript per million mapped reads (RPKM) for the *oy1* locus were obtained from a public repository of the National Science Foundation grant (GEPR: Genomic Analyses of shoot meristem function in maize; NSF DBI-0820610; https://ftp.maizegdb.org/MaizeGDB/FTP/shoot_apical_meristem_data_scanlon_lab/). These data consist of normalized read counts (expressed as RPKM) of the maize genes from the transcriptome of shoot apex of 14 days old IBM seedlings.

Marker data for Syn10 lines was obtained from Liu *et al.* (2015). The Syn10 lines were genotyped at 6611 positions (B73 RefGenv2) with SNP markers covering all ten chromosomes of maize. The entire set of markers were used for linkage analysis as there was no missing data. All B73-Mo17 NILs in both the B73 and Mo17 recurrent backgrounds that had introgression of the critical region from the opposite parent were selected for QTL validation. Genotyping data of the BM-NILs to choose informative lines and perform QTL validation was obtained from Eichten *et al.* (2011).

Genotypic data for the MDL used in this study to perform GWA were obtained from third generation maize haplotypes (HapMap3) described in Bukowski *et al.* (2018). Out of the 343 inbred lines that were used to develop F1 hybrids for phenotyping, only 305 lines were genotyped as part of HapMap3. The HapMap3 consists of over 83 million variant sites across ten chromosomes of maize that are anchored to B73 version 3 assembly. After obtaining these genotypic data, variant sites were filtered for ≤10% missing data and minor allele frequency of ≥0.05 using VCFtools (Danecek *et al.* 2011). This filtered SNP dataset was used for GWA analyses. A summary of the variant sites before and after the filtering procedure is in Table S5. The genotypic data for the set of 305 accessions were obtained from Romay *et al.* (2013). These genotypes consist of 681 257 SNPs (physical positions from B73 RefGenv2) obtained using a GBS protocol (Elshire *et al.* 2011) covering all ten chromosomes of maize. This marker dataset was filtered for ≤10% missing data and minor allele frequency of ≥0.05 using TASSEL (Bradbury *et al.* 2007), reducing the marker number to 150 920 SNPs. This genotypic dataset was solely used to compute principal components (PC) and a kinship matrix to control for population structure and familial relatedness in a unified mixed linear model, respectively (Yu *et al.* 2006). To test for *cis*-eQTL at the *oy1* locus in the maize diversity lines used for GWA mapping, normalized count of OY1 expression derived from the germinating seedling shoots was obtained from http://www.cyverse.org (Kremling *et al.* 2018).

### Statistical Analyses

QTL mapping: Line-cross phenotypes and markers were used to detect and localize QTL using the R/QTL package version 1.40-8 (Broman *et al.* 2003). Trait means were used for the QTL analyses. Single interval mapping (SIM) was used for all traits, although composite interval mapping (CIM) was carried out with remarkably similar results (data not shown). Statistical thresholds were computed by 1000 permutations for each trait (Churchill and Doerge 1994).

Genome-wide association study (GWAS): Preliminary data analysis was done using JMP 13.0. Statistical corrections on the raw phenotypic data were performed by determining the most significant terms in the model using analysis of variance (Fisher 1921). Genotype and replication were used as a random effect in a linear mixed-effects model built in the lme4 package (Bates *et al.* 2015) implemented in R (R Core 2014) to calculate the best linear unbiased predictor (BLUP) for each trait. Broad-sense heritability (line mean basis) were calculated using BLUP values, using the method described by Lin and Allaire (1977). BLUP estimates for each trait were used to perform GWAS. GWAS was done using a compressed mixed linear model implemented in the R package GAPIT (Zhang *et al.* 2010; Lipka *et al.* 2012). HapMap3 SNPs were used to calculate genotype to phenotype associations. As explained before, kinship and population structure estimates were obtained for the same population using the second subset of 150 920 SNPs to correct for spurious associations. The Bonferroni correction and false discovery rate (FDR) adjustments were used to compute a statistical threshold for the positive association to further control for false positive assessment of associations (Holm 1979; Benjamini and Hochberg 1995).

### Molecular Analyses

Genotyping: The recombinants in selected Syn10 lines and the BC1F1 population were detected using three PCR-based markers. Two markers detecting insertion polymorphisms flanking the *oy1* locus and one dCAPS marker at *oy1* locus were designed for this purpose. Genotyping at insertion-deletion (indel) marker *ftcl1* (flanking an indel polymorphism in intron 4 of *ftcl1*; GRMZM2G001904) was performed with forward primer 5’-GCAGAGCTGGAATATGGAATGC-3’ and reverse primer 5’-GATGACCTGAGTAGGGGTGC-3’. Genotyping at indel marker *gfa2* (flanking an indel polymorphism in the intron of *gfa2*; GRMZM2G118316) was performed with forward primer 5’-ACGGCTCCAAAAGTCGTGTA-3’ and reverse primer 5’-ATGGATGGGGTCAGGAAAGC-3’. A polymorphic SNP in the second intron at *oy1* was used to design a dCAPS forward primer 5’-CGCCCCCGTTCTCCAATCCTGC-3’ and a gene-specific reverse primer 5′-GACCTCGGGGCCCATGACCT-3′ using a web user interface at http://helix.wustl.edu/dcaps/dcaps.html (Neff *et al.* 2002). The PCR products from polymerization reactions with the dCAPS oligonucleotide at *oy1* were digested by *Pst*I restriction endonuclease (New England Biolabs, MA, USA) and resolved on 3.5% agarose gel.

Allele-specific expression analyses: Allelic bias at transcriptional level was quantified using the third leaf of maize seedlings at the V3 developmental stage. Total RNA was extracted using a modified Phenol/Lithium chloride protocol (Eggermont *et al.* 1996). Total RNA was subjected to DNase I treatment using Invitrogen Turbo DNA-free kit (Catalog# AM1907, Life Technologies, Carlsbad, CA) and 1 µg of DNase treated RNA from each sample was converted to cDNA using oligo dT primers and a recombinant M-MLV reverse transcriptase provided in iScriptTM Select cDNA synthesis kit (catalog# 170-8896, Bio-Rad, Hercules, CA) according to the manufacturer’s recommendations. Besides the cDNA samples, genomic DNA samples were also prepared as a control to test the sensitivity of the assay. Genomic DNA controls included a 1 1 (F1 hybrid), 1 2, and 2 1 mixture of B73 and Mo17. PCR was conducted using gene-specific forward primer 5’-CAACGTCATCGACCCCAAGA-3’ and reverse primer 5’-GGTTACCAGAGCCGATGAGG-3’ for 30 cycles (94° for 30s, 56° for 30s, 72° for 30s and final extension for 2 min) to amplify the OY1 gene product. These primers flank SNP252 (C->T), which is the causative mutation of *Oy1-N1989*, and SNP317 (C->T) which is polymorphic between B73 and Mo17 but monomorphic between the B73 and *Oy1-N1989* genetic backgrounds. Corresponding PCR products were used to generate sequencing libraries using transposon-mediated library construction with the Illumina Nextera DNA library preparation kit, and sequence data were generated on a MiSeq instrument (Illumina, San Diego, CA) at the Purdue Genomics Core Facility. The SNP variation and read counts were decoded from the sequenced PCR amplicons by alignment of the quality controlled reads to the *oy1* reference allele from B73 using the BBMap (Bushnell 2014) and the GATK packages (DePristo *et al.* 2011). Additional analysis was performed using IGV (Robinson *et al.* 2011) to manually quality-check the alignments and SNP calls. Read counts at polymorphic sites obtained from GATK was used to calculate allele-specific expression. Genomic DNA control samples showed bias in the read counts in a dosage-dependent manner. DNA from F1 hybrids between B73 and Mo17 resulted in 1:1 reads at *oy1* demonstrating no bias in the assay to quantify expression.

OY1 sequencing: The coding sequence of the *oy1* locus from B73, Mo17, W22, and *Oy1-N1989* homozygous seedlings were obtained from the genomic DNA using PCR amplification. For the rest of the maize inbred lines, the *oy1* locus was amplified from cDNA synthesized from total RNA derived from the shoot tissue of 14 days old maize seedlings. PCR amplification of the *oy1* locus from genomic DNA was performed using four primer pairs: (a) forward primer Oy1-FP1 5’-GCAAGCATGTTGGGCACAGCG-3’ and reverse primer Oy1-RP12 5’-GGGCGGCGGGATTGGAGAAC-3’, (b) forward primer Oy1-FP5 5’-GGTGGAGAGGGAGGGTATCT-3’ and reverse primer Oy1-RP6 5’-GGACCGAGGAAATACTTCCG-3’, (c) forward primer Oy1-F8 5’-ATGCCCCTTCTTCCTCTCCT-3’ and reverse primer Oy1-R8 5’-CGCCTTCTCGATGTCAATGG-3’, (d) forward primer Oy1-F9 5’-GGCACCATTGACATCGAGAA-3’ and reverse primer Oy1-R9 5’-GCTGTCCCTTCCTTTCAACG-3’. PCR amplification of OY1 transcripts from cDNA was performed using all primer pairs except Oy1-FP1/RP12. The PCR products from these samples were sequenced either using Sanger or Illumina sequencing. For Sanger sequencing, cleaned PCR products were used to perform a cycle reaction using Big Dye version 3.1 chemistry (Applied Biosystems, Waltham, MA) and run on ABI 3730XL sequencer by Purdue genomics core facility. Read with high-quality base pairs from Sanger sequencing were aligned using ClustalW (Thompson *et al.* 1994). Illumina sequencing was performed as described above except in this case paired-end reads were aligned to the B73 reference of *oy1* gene using bwa version 0.7.12 (Li and Durbin 2009) and variant calling was done using Samtools (Li *et al.* 2009).

### Data Availability

The public genotypic and expression datasets leveraged in this study are open access at the data repositories mentioned above. The input files containing genotypes and phenotypes of both line-cross populations that were used for QTL detection are provided in the supplemental section for re-analyses. All of the supplemental data (Figures S1-S9, Tables S1-S23, and File S1-S2) was deposited at figshare. The seed stocks described in this study are available upon request. Supplemental material available at Figshare: https://doi.org/10.25387/g3.7370948.

## Results

### Mo17 encodes an enhancer of the semi-dominant mutant allele *Oy1-N1989* of maize

The *Oy1-N1989* allele was recovered from a nitrosoguanidine mutant population in mixed genetic background. The molecular nature of the mutation is a single non-synonymous base pair change (Sawers *et al.* 2006). Heterozygous *Oy1-N1989* plants have the eponymous oil-yellow color but are reasonably vigorous and produce both ears and tassels. During introgression of the semi-dominant *Oy1-N1989* allele into B73 and Mo17 inbred backgrounds, we observed a dramatic suppression of the mutant phenotype in F1 crosses of the mutant stock (obtained from Maize COOP) to the B73 background. In contrast, crosses to Mo17 enhanced the mutant phenotype. The difference in phenotype expression was stable and persisted in both genetic backgrounds through all six backcross generations observed to date. To further explore and quantify this suppression, B73, Mo17, as well as *Oy1-N1989/oy1*:B73, crossed with each of these inbred lines were grown to the V3 stage in the greenhouse. To improve upon our visual assessment of leaf color and provide quantitation, optical absorbance was measured using a Chlorophyll Content Meter-200 plus (CCM; Opti-Sciences, Inc), a hand-held LED-based instrument. CCM is predicted to strongly correlate with chlorophyll and carotenoid contents. To confirm that our rapid phenotyping with the CCM would accurately assess chlorophyll levels, we measured the absolute pigment levels using a UV-VIS spectrophotometer (destructive sampling) on the same leaves used for CCM measurements. The non-destructive CCM measurements and the absolute pigment contents displayed a strong positive correlation with a R${}^{2}$ value of 0.94 for chl*a*, chl*b*, and total chlorophyll (Figure S1). Given this high correlation of maize leaf greenness between the rapid measurement using CCM-200 plus instrument and absolute pigment contents quantified using UV-VIS spectrophotometer, we performed all chlorophyll measurements of *Oy1-N1989* enhancement discussed in later results using CCM values.

In the greenhouse grown seedlings, chlorophyll accumulation was below the level of detection in the *Oy1-N1989* homozygotes using CCM and spectrophotometric method. In wild-type plants, CCM measurements were slightly higher in B73 than Mo17, but the spectrophotometric method did not identify any significant difference in the amount of chlorophyll *a* (chl*a*), chlorophyll *b* (chl*b*), total chlorophyll, or carotenoids between these two genotypes (Table S6). We detected a mild parent-of-origin-effect for both CCM and absolute amounts of chl*a*, chl*b*, total chlorophyll, and carotenoids in the wild-type siblings of our F1 crosses. These plants had slightly higher chlorophyll accumulation when B73 was used as the pollen parent (Table S6). However, no such effect of parent-of-origin was observed for the mutant heterozygotes (*Oy1-N1989/oy1*) and reciprocal hybrid combinations of crosses between *Oy1-N1989/oy1*:B73 and Mo17 were indistinguishable. Further, both CCM and absolute chlorophyll contents were higher in the *Oy1-N1989/oy1*:B73 plants compared to the mutants in B73 x Mo17 hybrid background. Thus, there was a strong effect of genetics on chlorophyll pigment variation in mutants, that went opposite to predictions for hybrid vigor.

**Figure 1:**
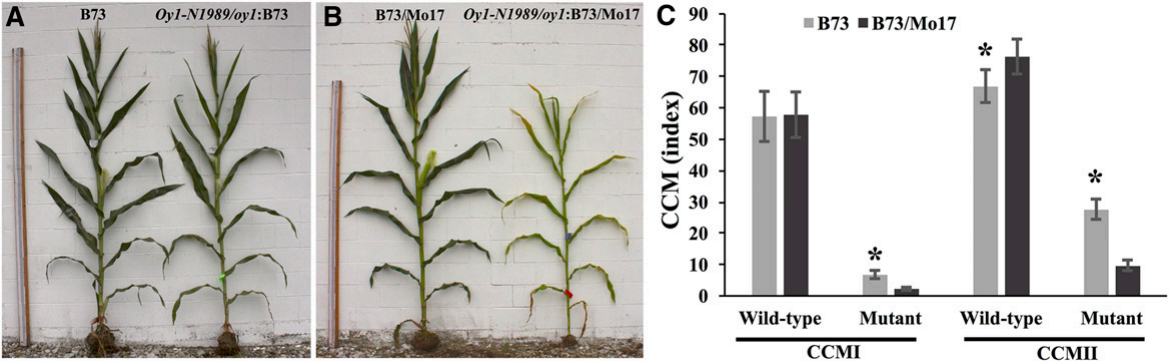
The chlorophyll pigment accumulation differs in the *Oy1-N1989/oy1* heterozygotes in the B73 and B73 x Mo17 hybrid backgrounds. Representative wild-type and mutant siblings from (A) B73 x *Oy1-N1989/oy1*:B73, and (B) Mo17 x *Oy1-N1989/oy1*:B73 F1 crosses in the field-grown plants. Measuring stick in panels A and B is 243 cm. (C) Non-destructive chlorophyll approximation in mutant and wild-type siblings at an ∼3 weeks (CCMI) and ∼6 weeks (CCMII) after planting; data for each class of genotype is derived from 39 replications planted in RCBD. Asterisks indicate statistical significance between the means in each genotypic category at *P* < 0.01 using student’s *t*-test.

We tested the progenies from the crosses of *Oy1-N1989/oy1*:B73 with B73 and Mo17 inbred lines in the field. Consistent with our previous observation, mutant heterozygotes in the B73 inbred background were substantially greener than heterozygotes in the Mo17 x B73 F1 hybrid background (Figure 1, Table S7). The increased severity of *Oy1-N1989* heterozygotes in the Mo17 genetic background was stable across generations and observed even after six backcrosses (Figure S2). No suppressed mutant plants were observed during any generation of backcrossing into Mo17 (data not shown). Thus, the expected negative impact of the *Oy1-N1989* allele on chlorophyll pigment accumulation was dramatically suppressed by the B73 background compared to Mo17 genotype.

### vey1 maps to a single locus that co-segregates With the oy1 allele of Mo17 in DH, RIL, BC1F1 and NIL families derived From B73 and Mo17

**Figure 2:**
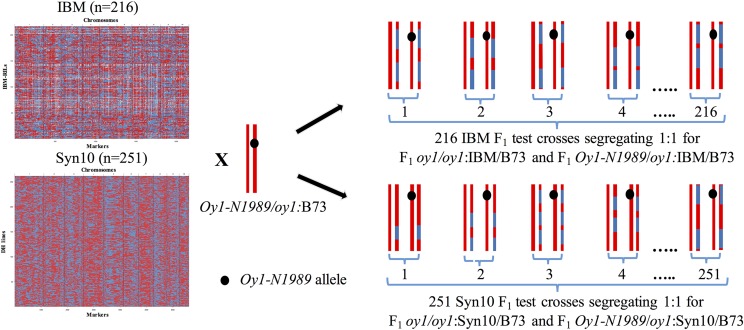
The crossing scheme used to map *Oy1-N1989* enhancer/suppressor loci in IBM and Syn10 populations. Red, blue and white colors indicate B73, Mo17, and missing genotypes, respectively. The heterozygous tester shows chromosome 10 with a black spot indicating *Oy1-N1989* mutant allele. F1 progenies depicted here shows hypothetical genotypes of chromosome 10 for each F1 wild-type and mutant siblings.

**Figure 3:**
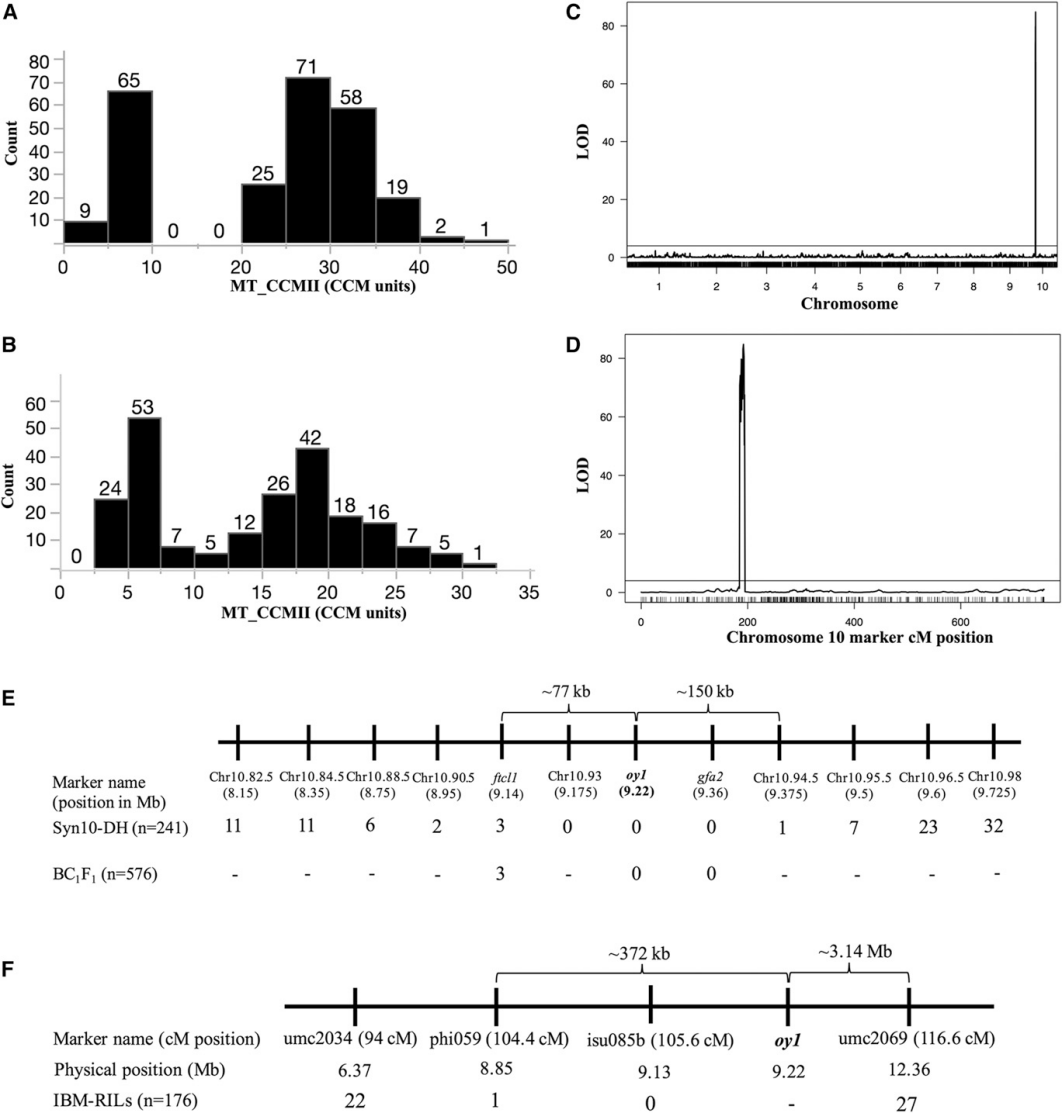
The phenotypic distribution, QTL analysis, and fine mapping results of MT_CCMII. Distribution of MT_CCMII in (A) Syn10 x *Oy1-N1989/oy1*:B73, and (B) IBM x *Oy1-N1989/oy1*:B73 F1 populations. (C) Genome wide QTL plot of MT_CCMII in Syn10 x *Oy1-N1989/oy1*:B73 F1 population. (D) Close-up view of the *vey1* locus on chromosome 10. Black horizontal bar in panels C-D indicate permutation testing based threshold to declare statistical significance of the QTL. Recombinants detected at *vey1* in F1 crosses of *Oy1-N1989/oy1*:B73 with (E) Syn10, B73 x Mo17 F1 (BC1F1), and (F) IBM. Number at a given marker and population intersection in (E) and (F) indicates the total number of recombinants between the respective marker genotype and observed MT_CCMII phenotype; hyphen denotes no genotyping. dCAPS marker at *oy1* is highlighted in bold.

To identify the genetic basis of the suppression of *Oy1-N1989* allele in B73, we performed a series of crosses to four mapping populations. In each case, we crossed pollen from heterozygous *Oy1-N1989* plants to a population of recombinant lines as ear-parents (Figure 2). We chose two public populations, IBM and Syn10, to map all modifiers altering the severity of the *Oy1-N1989* phenotypes. IBM and Syn10 differ in the number of rounds of intermating, and therefore vary in the number of recombinants captured and genetic resolution of trait localization (Lee *et al.* 2002; Hussain *et al.* 2007). Each F1 progeny of the testcross segregated approximately 1:1 for wild-type (*oy1/oy1*) and mutant heterozygote (*Oy1-N1989/oy1*) in the hybrid genetic background with B73. Mutant heterozygote siblings in both (IBM and Syn10) F1 populations, chlorophyll approximation using CCM measured at an early (CCMI) and late (CCMII) developmental stages displayed bimodal trait distributions (Figures 3A and 3B; Figures S3 and S4), suggesting the presence of an allele of strong effect. The CCM distributions of the wild-type and mutant siblings did not overlap (Figures S5A and S5B). CCM values collected at both time points in the wild-type F1 siblings showed positive correlations in both F1 populations (Tables S8 and S9; Figures S3 and S4). A similar trend was also observed for the CCMI and CCMII in the mutant F1 siblings. The CCM measurements in the wild-type and mutant siblings did not display any significant correlation. To control for variation in CCM observed due to the genetic potential of each line that was independent of the *Oy1-N1989* modification, we divided the mutant CCM trait values by the congenic wild-type sibling CCM values to derive ratio for both time points. We also calculated differences between congenic wild-type and mutant CCM values. Each of the direct measurements, as well as the ratio-metric and difference values, were used as phenotypes to detect and localize QTL.

Summary of the peak positions of all QTL passing a permutation computed threshold are presented in Table S10 for the IBM F1 populations and Table S11 for the Syn10 F1 populations. All mutant CCM traits, all mutant to wild-type CCM ratios, and all differences between mutant and wild-type CCM measurements identified a single QTL of large effect on chromosome 10. We name this suppressor locus *very oil yellow1* (*vey1*). A plot of the log10 of odds (LOD) score with permutation calculated threshold for CCMII from the mutant siblings in the Syn10 F1 population is plotted in Figures 3C and 3D. Other mutant-related traits in Syn10 and IBM F1 populations produced similar plots (data not shown). One additional minor effect QTL was identified from these data, and it only influenced the early chlorophyll measurements in wild-type siblings of the IBM F1 population (Table S10). Interestingly, *vey1* mapped to the same genetic position as *oy1* locus itself (Figures 3E and 3F) and there was no influence of this locus on CCM traits in wild-type siblings. These analyses indicate that the *vey1* QTL encodes a single locus with an effect contingent upon the presence of the *Oy1-N1989* allele.

The high penetrance of the MT_CCMII trait allowed us to interpret a discordance between the marker genotype and F1 mutant phenotype of high or low CCM categorization as recombination between *vey1* and the given marker. We classified each F1 mutant hybrid in the Syn10 and IBM F1 populations as high or low CCMII using the bimodal distribution. We then compared the marker genotypes at each marker linked to *vey1* with the phenotypic categories. Table S12 shows the mutant trait values, marker genotypes, and phenotypic categories for the nine F1 Syn10 lines with recombinants within the *vey1* region (between the flanking markers *10.90.5* and *10.95.5*). A summary of the recombinants between the markers and the phenotypic class at each marker position across the *vey1* region is presented in Figures 3E and 3F. Genotypes at marker *10.93* perfectly predicted CCMII trait expression in Syn10 F1 mutant siblings and recombinants placed this QTL within the ∼227 kb interval between markers *10.94.5* and *10.90.5* (Figure 3E). This *vey1* region includes the *oy1* locus itself, suggesting that the Mo17 allele of *oy1* may enhance the impact of the *Oy1-N1989* allele. Three Syn10 DH lines recombinant within this critical region were genotyped with additional markers developed at *ftcl1* (GRMZM2G001904), and *gfa2* (GRMZM2G118316). Three recombinants were detected between *vey1* and *ftcl1* but no recombinants were recovered between the *gfa2* indel marker and *vey1*. A SNP marker used in the Syn10 map construction (10.94.5) at the distal end of the *gfa2* locus identified one recombinant (Figure 3E). Thus, *vey1* is localized between *ftcl1* and *gfa2*. This region includes *oy1*, *ereb28*, a small region of *gfa2* (from marker *10.94.5*), and *ftcl1* proximate to *oy1*. A similar fine-mapping attempt with the IBM F1 population provided no additional resolution (Figure 3F). The genotyping of 576 BC1F1 individuals from (B73 x Mo17) x *Oy1-N1989/oy1*:B73 crosses failed to recover any recombinants between *vey1* and *gfa2* (Figure 3E).

We further explored background influence on the *vey1* alleles from B73 and Mo17 using a series of BM-NILs that contained the *vey1* region introgressed into a homogeneous background of either B73 or Mo17. Progeny from crosses of BM-NILs with *Oy1-N1989/oy1*:B73 displayed the expected mutant CCM phenotype based on the *vey1* introgression regardless of the recurrent parent (Figure S6 and Table S3). These data confirm the expectation of the QTL mapping but offered no additional recombinants within the identified *vey1* QTL.

Thus, the formal list of candidate genes for the *vey1* QTL is the *oy1* gene itself and the three most closely linked loci: *ftcl1*; *gfa2*; *ereb28*. The ortholog of *Zmftcl1* (∼62% protein identity) from *Arabidopsis thaliana* is required for folate metabolism (Pribat *et al.* 2011). The maize gene *gfa2* is uncharacterized, but mutation of the *Arabidopsis* ortholog caused defects in megagametogenesis including failures of polar nuclear fusion in the female gametophyte and synergid cell-death at fertilization (Christensen *et al.* 1998, 2002). The third linked gene, *ereb28* (*Apetela2-Ethylene Responsive Element Binding Protein-transcription factor 28*) has a highly conserved AP2/EREB domain. This gene has a very low expression level and is localized only to the root tissue of maize (https://www.maizegdb.org/gene_center/gene?id=GRMZM2G544539#rnaseq).

### Controlling for the vey1 QTL did not reveal additional modifiers of Oy1-N1989 phenotype expression

The non-normality of some of the trait distributions and apparent thresholds prompted us to explore additional QTL models. No additional QTL were recovered by implementing two-part threshold models (Broman *et al.* 2003) for any of the traits (data not shown). Similarly, two-way genome scans also failed to detect any statistically significant genetic interactions with *vey1* or between other loci. In both the IBM and the Syn10, the region encoding *vey1* exhibited substantial segregation distortion with the B73:Mo17 alleles present at 120:72 in the IBM and 175:76 in the Syn10 population. This uneven sample size will reduce the power to detect epistasis with *vey1* but would not limit the detection of additional unlinked epistatic modifiers of *Oy1-N1989*.

We used the top marker at *vey1* as a covariate to control for the contribution of this allele to phenotypic variation and performed a one-dimensional scan of the genome (Broman *et al.* 2003). In our previous naïve one-dimensional scans, the large effect of *vey1* partitioned into the error term and might reduce our power to detect additional unlinked QTL(s). By adding a marker linked to *vey1* as a covariate, this term will capture the variance explained by *vey1* and could improve detection of additional QTL(s) of presumably smaller effect. In both populations, use of a *vey1* linked marker as a covariate did not identify any additional QTL for any trait (data not shown). Thus, modification of the *Oy1-N1989* phenotype by *vey1* was inherited as a single QTL, acting alone.

### GWAS for chlorophyll content in maize diversity lines (MDL) and Oy1-N1989/oy1 F1 genotypes identifies vey1

We undertook GWA mapping of *Oy1-N1989* severity to search for additional loci and potentially identify recombinants at *vey1*. A population of 343 lines was crossed to *Oy1-N1989/oy1*:B73. This generated MDL x *Oy1-N1989/oy*1:B73 F1 populations segregating 1:1 for mutant and wild-type siblings in hybrid genetic background. There was total separation between mutant and wild-type siblings for the CCMI and CCMII traits (Figure S5C). The mutant severity of some F1 families was similar to the F1 progeny of Mo17 x *Oy1-N1989/oy1*:B73 (Figure S7). Pairwise correlations of the CCM trait measurements at two time points (CCMI and CCMII) in the wild-type siblings displayed statistically significant positive relationship (Table S13). CCM traits were much more strongly correlated in the mutant F1 siblings, similar to the B73 x Mo17 F1 populations. However, weak positive correlations were also observed between mutant and wild-type CCM measurements in the MDL F1 populations. Broad-sense heritability estimates for CCMI and CCMII were high for the mutant and ratio traits, whereas wild-type siblings had much lower repeatability (Table S14).

**Figure 4:**
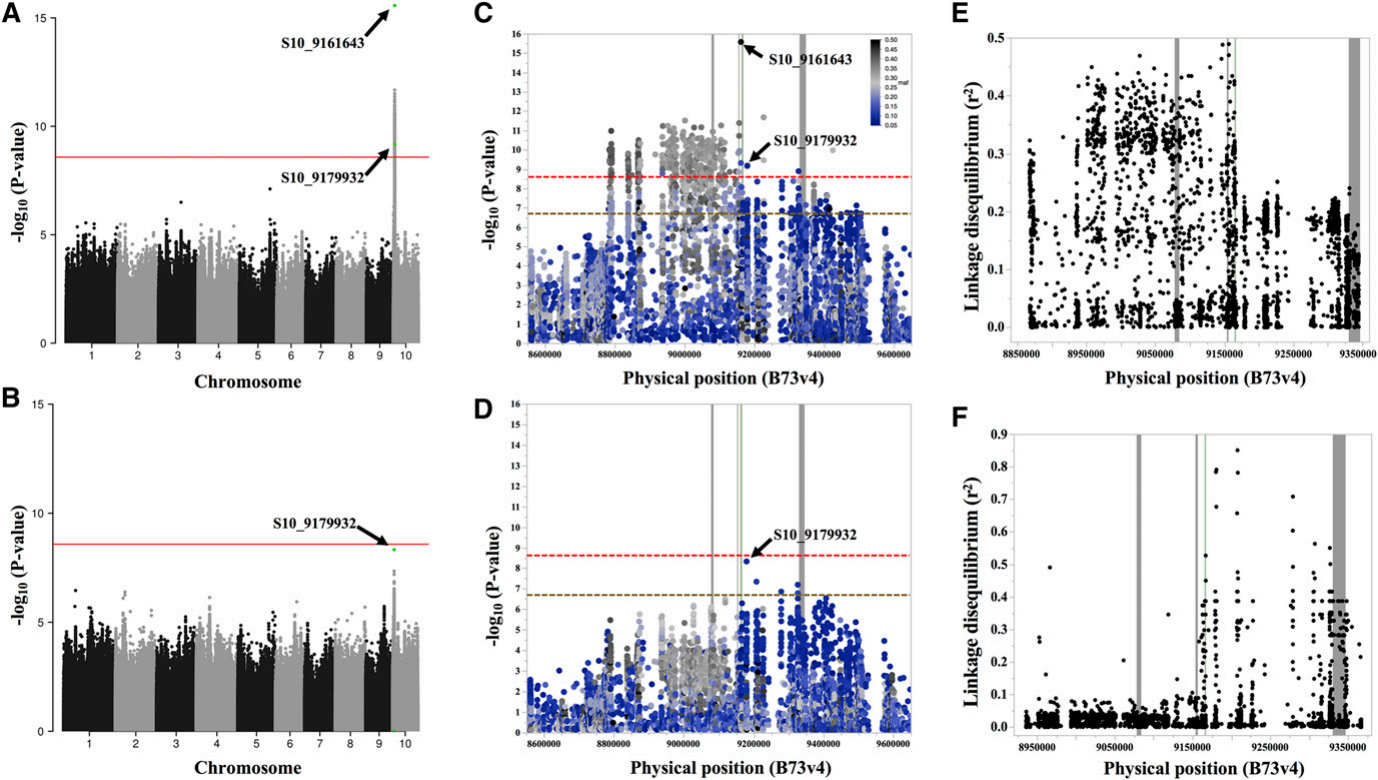
The Manhattan plots of SNP associations with MT_CCMII in MDL x *Oy1-N1989/oy1*:B73 F1 population. Genome-wide association of (A) MT_CCMII with no marker covariate, (B) MT_CCMII using S10_9161643 as a covariate. Close-up views of the SNP associations in the *vey1* region on chromosome 10 for (C) Panel A, (D) Panel B. Arrows in panels A-D identify SNPs S10_9161643 and S10_9179932. Horizontal solid red and hashed red lines in panels A-D indicate the genome-wide Bonferroni cut-off at *P* < 0.05, and hashed golden line in panels C-D is a chromosome-wide FDR-adjusted threshold at *P* < 0.05. Linkage disequilibrium of all SNPs in a 450 kb region flanking *oy1* with SNPs (E) S10_9161643, and (F) S10_9179932. Vertical lines in panels C-F from left to right represent genomic position of *ftcl1*, *ereb28*, *oy1* (green), and *gfa2*.

GWA was performed using the 305 inbred lines from MDL crosses that were present in the HapMap3 SNP data set (Bukowski *et al.* 2018). The variation in MT_CCMI, MT_CCMII, and their ratios identified a region encoding multiple SNPs that passed a multiple test correction (see Methods) on chromosome 10 at the site of the *oy1* gene. No other loci passed the corrected threshold in the GWA analysis. No statistically significant SNPs were detected for the CCM traits of wild-type siblings. The plot showing the negative log10 of the p-values from GWA tests for all the SNPs for MT_CCMII trait is graphed in Figure 4A. A closer view of the SNPs within the region encoding the *vey1* locus on chromosome 10 are plotted in Figure 4C. A summary of the GWAS results for mutant CCM and ratio traits is presented in Table S15. The top association for the mutant CCM traits was a SNP at position 9161643 on chromosome 10 (S10_9161643) located just 3’ of the *oy1* protein-coding sequence. S10_9161643 displays high allelic frequency in our population (f = 0.49). Thus, it appears that variation at the *oy1* locus may be responsible for the suppression of the *Oy1-N1989* mutant allele in the diverse panel of maize inbred lines analyzed in this experiment. Analysis of the LD between S10_9161643 and the other variants in this region identified no other variants with r${}^{2}$ greater than 0.5 despite the relatively strong associations between many SNPs and the CCM traits (Figure 4E). LD was substantially higher for SNPs encoded toward the telomere from *oy1* than toward the centromere, with a strong discontinuity of LD at the 3’-end of *oy1*. Given the relatively low p-values calculated for multiple SNPs in the area, this raises the possibility that multiple alleles contribute to the suppression of the *Oy1-N1989* phenotype. To test this hypothesis, the genotypes at SNP S10_9161643 were used as a covariate, and the genetic associations were recalculated. If SNPs segregate independently of S10_9161643 and contribute to *Oy1-N1989* suppression, the p-values of association test statistics should stay significant. On the contrary, those SNPs that have relatively low p-values due to linkage with S10_9161643 should become statistically insignificant in the covariate model. When these analyses were done, low-frequency variants at the 5’ end of the *oy1* locus were identified as the most significant SNPs and passed a chromosome-wide multiple test correction (Figure 4B and 4D). This result suggests that there are multiple alleles capable of modifying the *Oy1-N1989* mutant phenotype in the MDL panel. The top SNP on chromosome 10 in the covariate model of MLM was detected at position 9179932 (S10_9179932), and the allele associated with *Oy1-N1989* suppression is a relatively low-frequency variant (f = 0.08). It remains formally possible that the SNP S10_9179932 is not causative and merely in LD with a causative polymorphism, and the second locus is fortuitously present in recombinant haplotypes. Analysis of LD of S10_9179932 with other SNPs in ∼500 kb window detected multiple SNPs of low allelic frequency that were in high LD (r${}^{2}$ ∼0.85) toward the 5’ end of *oy1* (Figure 4F). Consistent with the strong discontinuity of LD at 3’ end of *oy1* with S10_9161643, we observed discontinuity of LD with S10_9179932 at 5’ end of the *oy1* locus. The analyses suggest that S10_9161643 and S10_9179932 are not in LD with each other and can act independently. The MLM model that considered only these two SNPs accounted for ∼29% of the variation in CCMII measurements of the mutant siblings (Table S15).

Given that the MLM model using S10_9161643 as a covariate detected S10_9179932 as the most significant association, we tested the phenotypic outcome of the four possible haplotypes at these two SNPs. We observed that the four haplotypes varied only for mutant CCM traits, with haplotypes AG and CA being the most favorable (highest CCM mean) and least favorable (lowest CCM mean), respectively (Table S16). Alleles at these two SNPs affected CCMI and CCMII in the mutant plants, consistent with additive inheritance for two polymorphisms. The additive effect of the SNPs is consistent with independent *cis*-regulatory alleles at *oy1* modifying the *Oy1-N1989* mutant phenotype. Consistent with the strong enhancement caused by crossing Mo17 to *Oy1-N1989/oy1*:B73, the Mo17 *oy1* locus encodes the most severe, and relatively rare, CA allele combination, and B73 encodes the most suppressing AG allele combination (Table S16). Thus, the line-cross mapping was performed with inbred lines that carry phenotypically extreme allele combinations of these two SNPs in the vicinity of the *oy1* locus.

### Oy1-N1989 is a semi-dominant chlorophyll mutant and enhanced by reduced function at oy1

**Figure 5:**
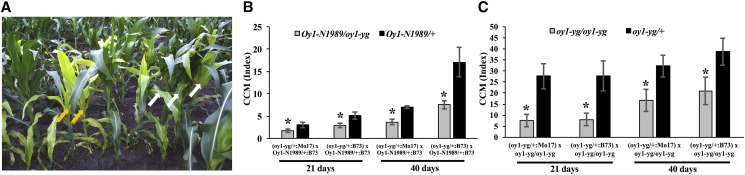
The single locus test of *oy1* showing the interaction between wild-type alleles of *oy1* from B73 and Mo17 with the semi-dominant (*Oy1-N1989*), and recessive mutant allele (*oy1-yg*) at *oy1*. (A) Mutant (two severity groups) and wild-type individuals segregating in a cross (B73 x *oy1-yg/oy1-yg*) x *Oy1-N1989/+*:B73. White-fill arrows indicate *Oy1-N1989/+* plants (pale-green and suppressed), whereas yellow-fill arrows indicate *Oy1-N1989/oy1-yg* (yellow-green and severe) plants. CCM measurements of the test crosses at 21 and 40 days after planting in the (B) Mutant siblings (*Oy1-N1989/oy1-yg* and *Oy1-N1989/+*) of (Mo17 x *oy1-yg/oy1-yg*) x *Oy1-N1989/+*:B73, and (B73 x *oy1-yg/oy1-yg*) x *Oy1-N1989/+*:B73 crosses, (C) Mutant (*oy1-yg/oy1-yg*) and wild-type (*oy1-yg/+*) siblings of (Mo17 x *oy1-yg/oy1-yg*) x *oy1-yg/ oy1-yg* and (B73 x *oy1-yg/oy1-yg*) x *oy1-yg/ oy1-yg* crosses. Asterisk in panels B-C indicate statistically significant difference between the genotypes in a given cross at *P* < 0.01 using student’s *t*-test.

Maize seedlings that are double heterozygotes for *Oy1-N1989* and hypomorphic *oy1* allele (*chlI-MTM1*) are more severe than isogenic *Oy1-N1989/oy1* siblings (Sawers *et al.* 2006). To confirm that the reduced *oy1* function could determine the differential sensitivity to *Oy1-N1989*, we crossed dominant and recessive mutant alleles of *oy1* to each other. The recessive weak hypomorphic allele *oy1-yg* was obtained from the Maize COOP in the unknown genetic background. The homozygous *oy1-yg* plants were crossed as a pollen-parent with both B73 and Mo17 to develop F1 material that would segregate the mutation. The F1 plants were then crossed to *Oy1-N1989/oy1*:B73 as well as backcrossed to the *oy1-yg* homozygotes in the original mixed background. These crosses allowed us to recover plants that had *Oy1-N1989* in combination with the wild-type *oy1*-B73, wild-type *oy1*-Mo17, and mutant *oy1-yg* alleles. Chlorophyll contents were determined using CCM at 21 and 40 days after sowing in the field. The *Oy1-N1989* allele was substantially enhanced when combined with the *oy1-yg* allele, demonstrating that reduced function of the *oy1* allele in Mo17 could be the genetic basis of *vey1* QTL (Figures 5A and 5B). A summary of these data are presented in Table S17. This result is similar to the one described by Sawers *et al.* (2006), where a reduction in chlorophyll content was observed when *Oy1-N1989* allele was combined with a recessive allele of *oy1*. We also noticed a similar drop in chlorophyll accumulation in the *oy1-yg* homozygotes as oppose to *oy1-yg* heterozygotes with wild-type *oy1* allele from both B73 and Mo17 in the BC1F1 progenies (Figure 5C and Table S17). However, we did not observe any significant difference in the *oy1-yg* heterozygotes with B73 and Mo17 wild-type *oy1* allele. Selfed progeny from *Oy1-N1989* heterozygotes segregated for yellow-seedling lethal *Oy1-N1989* homozygotes with no detectable chlorophyll by either CCM or spectrophotometer quantification (Table S6). Therefore, consistent with previous work (Hansson *et al.* 2002; Sawers *et al.* 2006), the *Oy1-N1989* is a dominant-negative neomorphic mutant allele with no evident MgChl activity under the tested conditions. Based on these genetic data, any QTL resulting in decreased expression of *oy1* or an increased proportion of mutant to wild-type gene product in the *Oy1-N1989/oy1* heterozygotes can increase the severity of the mutant phenotype.

### No coding sequence difference in OY1 accounts for vey1 inheritance

**Figure 6:**
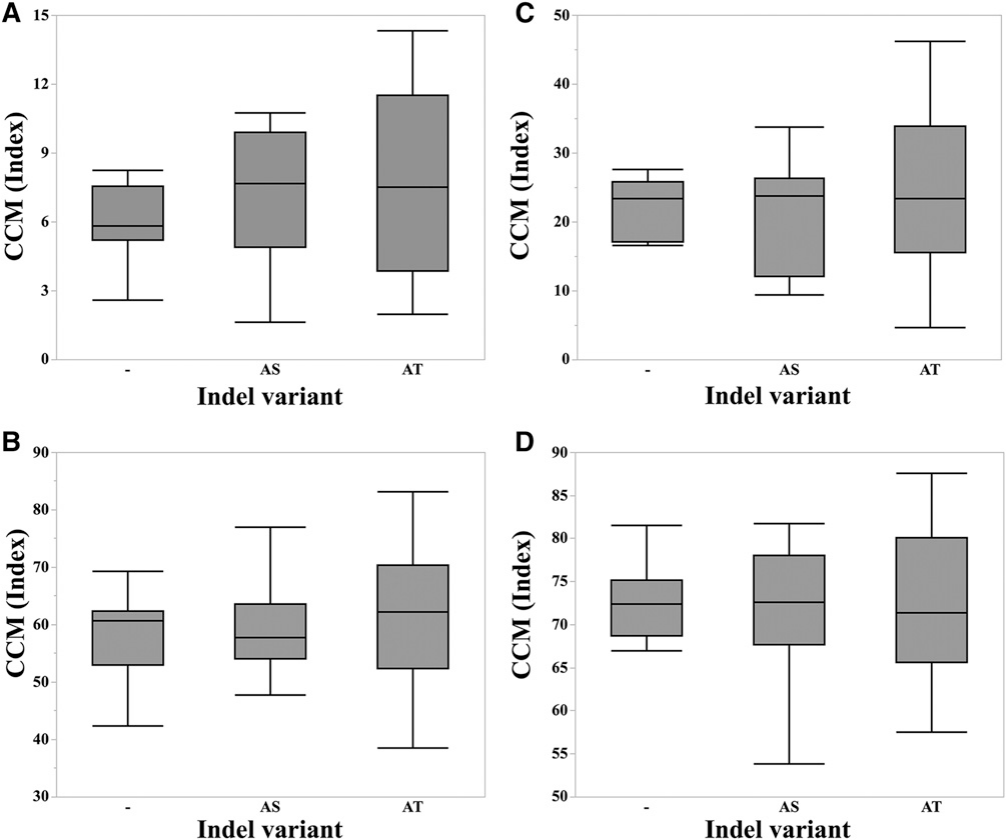
The distributions of CCM trait measurements in the F1 progenies of a sub-set of maize inbred lines crossed with *Oy1-N1989/oy1*:B73 divided using three allelic variants in the *oy1* coding sequence of the respective inbred line. Phenotypic distribution of (A) MT_CCMI, (B) WT_CCMI, (C) MT_CCMII, and (D) WT_CCMII. Symbols “-“, “AS”, and “AT” on the X-axis of each panel denote deletion of 6 base pairs (bp), insertion of amino acid residues Alanine-Serine (AS), and Alanine-Threonine (AT), respectively, in a given inbred line. Three inbred lines including B73 carried “-“ allele, six inbred lines carried “AS” insertion, nine inbred lines carried “AT” insertion. No statistically significant differences were found among three categories using ANOVA.

Our reliance on SNP variation leaves us open to the problem that linked, but the unknown non-SNP variation can be responsible for *vey1*. Given that reduced *oy1* activity enhanced the phenotype of *Oy1-N1989*, we sequenced the *oy1* locus from Mo17 and B73 to determine if coding sequence differences could encode the *vey1* modifier. The only non-synonymous changes that distinguish these two alleles is at the site of the previously reported in-frame 6 bp insertion (Sawers *et al.* 2006), which adds alanine (A) and threonine (T) amino acid residues to the OY1 protein. PCR amplification of *oy1* locus in 18 maize inbred lines, as well as the *Oy1-N1989* allele, was performed. Sequencing of the PCR products confirmed the absence of the 6 bp insertion in *Oy1-N1989* allele reported by Sawers *et al.* (2006). In addition, multiple inbred lines including B73, CML103, and CML322 also carried this 6 bp in-frame deletion. A polymorphism within the 6 bp insertion was also found that resulted in an alternative in-frame insertion encoding an alanine and serine (S) codon in Mo17 and five other inbred lines. Thus, three alleles at this site were found to be a common variant in OY1 gene product. These allelic states of *oy1* did not explain the phenotypic severity of CCM trait value in the F1 mutant siblings (Figure 6). The allelic state of *oy1* at this polymorphic site in 18 maize inbred lines and the average CCM trait values in the wild-type and mutant siblings of their respective F1 progenies with *Oy1-N1989/oy1*:B73 are summarized in Table S18. Five inbred lines, including Mo17, resulted in dramatic enhancement of the mutant CCMI and ratio of CCMI phenotypes of F1 plants crossed to *Oy1-N1989/oy1*:B73. These enhanced genotypes encoded all three possible alleles at *oy1*. In addition, the suppressing inbred lines also encoded all three possible alleles. Besides this 6 bp indel, three inbred lines had few more variants in OY1 protein. An enhancing inbred line CML322 had two missense mutations that lead to amino acid change at position 321 (D->E), and 374 (S->I). A suppressing inbred line NC358 had one amino acid change at position 336 (D->G) and the enhancing inbred Tzi8 had a 15 bp in-frame deletion leading to the removal of five amino acids (VMGPE) in the third exon of the coding sequence. Even considering the additional alleles at *oy1* found in few maize inbred lines, these results suggest that the only coding sequence polymorphism at *oy1* between B73 and Mo17 could not be the genetic basis of *vey1*. This result leaves the two additive top SNPs *in cis* with *oy1* as the most likely cause of *cis*-acting regulatory variation.

### Expression level polymorphism at oy1 co-segregates with suppression of Oy1-N1989 mutant phenotype

**Figure 7:**
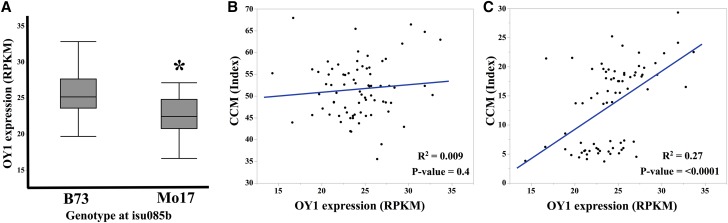
Expression of OY1 in the shoot apices of 14 days old IBM seedlings co-segregates with marker linked to *vey1*. (A) Distribution of OY1 RPKM values (X-axis) in the IBM RILs using the marker genotype at *isu085b* (linked to *oy1* and *vey1*). Asterisk indicate significant difference in the mean between two groups using student’s *t*-test at *P* < 0.01. Linear regression of OY1 expression in IBM RILs on CCMII in the (B) Wild-type and (C) Mutant siblings derived from IBM x *Oy1-N1989/oy1*:B73 crosses.

Measurements of mRNA accumulation from *oy1* in the IBM was publicly available in a previously published study (Li *et al.* 2013, 2018). Out of 105 IBM lines that were assessed for expression level in the past study, 74 were among those tested for chlorophyll accumulation in the *Oy1-N1989* F1 hybrid populations. Using a genetic marker linked to the *oy1* locus, we determined that a *cis*-acting eQTL controlled the accumulation of OY1 transcripts in IBM shoot apex (Figure 7). A summary of these data are presented in Table S19. This *cis*-acting eQTL conditioned greater expression of the B73 allele and explained 19% variation in OY1 transcript abundance in the IBM. Given the enhancement of *Oy1-N1989* by the *oy1-yg* allele, a lower expression of the wild-type *oy1* allele from Mo17 is expected to enhance the phenotype of *Oy1-N1989* (Figure 5). In addition, OY1 RPKM values obtained from the shoot apex of IBM were able to predict the CCM trait values in the mutant but not the wild-type siblings in IBM F1 population with *Oy1-N1989/oy1*:B73 (Figure 7). This result suggests that inbred lines with increased MgChl subunit I transcripts available for protein production and MgChl complex assembly could overcome chlorophyll accumulation defects caused by the *Oy1-N1989/oy1* genotype in IBM F1 hybrids. Consistent with this, a full linear regression model that included both the isu085b marker (*cis*-eQTL) genotypes and the residual variation in RPKM at OY1 did a better job in predicting CCMI and CCMII in the IBM mutant F1 hybrids than the isu085b marker by itself. If the *cis*-eQTL at *oy1*, which results in differential accumulation of OY1 transcripts in the IBM inbred lines can affect allele-specific expression in the F1 hybrids, it could explain the better performance of the IBM mutant F1 hybrids with the B73 allele at *vey1*.

A previous study of allele-specific expression in the F1 hybrid maize seedlings identified expression bias at *oy1* toward B73 in the hybrid combinations of B73 inbred line with PH207 and Mo17 but not Oh43 (Waters *et al.* 2017). We used two SNP positions, SNP_252 and SNP_317, to explore the allele-specific expression of OY1 in our materials. SNP_252 is the causative polymorphism for the *Oy1-N1989* missense allele while SNP_317 is polymorphic between B73 and Mo17, but monomorphic between *Oy1-N1989* and B73. As the original allele of the *Oy1-N1989* mutation was isolated from a *r1 c1* colorless synthetic stock of mixed parentage, this raises the possibility that the same *cis*-acting regulatory variation that lowered expression of OY1 from PH207 and Mo17 when combined with the B73 allele might also be present in the *oy1* allele that was the progenitor of *Oy1-N1989*. We tested this possibility by using the SNPs that distinguish B73, Mo17, and the *Oy1-N1989* alleles to measure allele-specific expression in each of the hybrids. Consistent with the previous data (Waters *et al.* 2017), we observed a biased expression at *oy1* toward the B73 allele in the B73 x Mo17 F1 wild-type hybrids (Table 1). Extended data from this experiment is provided in Table S20. In the B73 isogenic crosses, transcripts from the *Oy1-N1989* and B73 wild-type alleles accumulated to equal levels in the heterozygotes, indicating that the suppressed phenotype of the mutants in B73 background was not due to a lowered expression of *Oy1-N1989* relative to the wild-type allele. Remarkably, mutant siblings from the reciprocal crosses between *Oy1-N1989/oy1*:B73 and Mo17 resulted in greater expression from the *Oy1-N1989* allele than the wild-type *oy1* allele of Mo17. Allele-specific bias at *oy1* was significantly higher toward the *Oy1-N1989* allele in the *Oy1-N1989* mutant heterozygotes in the B73 x Mo17 hybrid background compared to B73 isogenic material. Thus, in mutant hybrids, overexpression of *Oy1-N1989* relative to the wild-type *oy1* allele in Mo17 could account for increased phenotypic severity.

**Table 1:** The allele-specific expression at *oy1* in the top fully-expanded leaf at the V3 developmental stage of B73 x Mo17 F1 wild-type and *Oy1-N1989/oy1* mutant siblings, and inbred *Oy1-N1989/oy1*:B73 mutants

Genotype	SNP_252	SNP_317	Ratio_SNP252*^a^*	Ratio_SNP317*^a^*	Average*^a^*
*oy1/oy1*:B73/Mo17*^b^*	.	.	.	.	1.19 ± 0.07
*oy1/oy1*:B73/Mo17	C/C	C/T	.	1.08 ± 0.01*^a^*	1.08 ± 0.07*^a^*
*Oy1-N1989/oy1*:Mo17/B73	C/T	C/T	1.12 ± 0.01*^a^*	1.10 ± 0.02*^a^*	1.11 ± 0.01*^a^*
*Oy1-N1989/oy1*:B73/Mo17	C/T	C/T	1.15 ± 0.03*^a^*	1.10 ± 0.01*^a^*	1.13 ± 0.02*^a^*
*Oy1-N1989/oy1*:B73	C/T	C/C	1.01 ± 0.02*^b^*	.	1.01 ± 0.02*^b^*

a The mean ± SD of the ratios of the read count from the reference/alternate allele at SNP_252, SNP_317, and the average of the ratios at SNP position 252 and 317. The connecting letter report indicates the statistical significance calculated using ANOVA with post-hoc analysis using Tukey’s HSD with *P* < 0.01.

b Data obtained from Waters *et al.* (2017).

If *vey1* is encoded by an eQTL, then PH207 should encode an enhancing allele and Oh43 should encode a suppressing allele of *vey1*. We tested this genetically by producing F1 progenies in crosses of PH207 and Oh43 by *Oy1-N1989/oy1*:B73 pollen. Oh43 was evaluated in our initial screening and also as part of the MDL panel used for GWAS. In both experiments, the F1 hybrids between Oh43 and *Oy1-N1989/oy1*:B73 suppressed the mutant phenotype, suggesting that Oh43 is a suppressing inbred line (CCM values in Tables S4 and S18). B73 x PH207 F1 hybrids were missing from our previous datasets. We crossed PH207 ears with pollen from *Oy1-N1989/oy1*:B73 plants. The F1 hybrids from this cross were analyzed in the greenhouse at seedlings stage along with F1 hybrids of B73 x *Oy1-N1989/oy1*:B73 and Mo17 x *Oy1-N1989/oy1*:B73 F1 progenies as controls. PH207 was an enhancing inbred genotype as mutant heterozygotes in a PH207 x B73 F1 genetic background accumulated less chlorophyll than mutants in the B73 isogenic background (Figure S8 and Table S21).

We further leveraged the normalized expression data of OY1 in the emerging shoot tissue of the maize diversity lines (Kremling *et al.* 2018) and used the top two additive SNPs (S10_9161643 and S10_9179932) at *vey1* from GWAS to test if these *cis*-variants of *oy1* affect its expression. Alleles that suppress *Oy1-N1989* linked to either SNP were associated with the greater abundance of OY1 transcripts. Plants carrying the B73-like allele combination (A and G at marker S10_9161643 and S10_9179932, respectively) showed the highest OY1 abundance in the emerging shoots of diverse maize inbred lines, whereas, plants with the Mo17-like allele combination (C and A at S10_9161643 and S10_9179932, respectively) showed lowest OY1 count (Table S22). Consistent with the additive suppression of leaf greenness in *Oy1-N1989* mutants by the alleles at S10_9161643 and S10_9179932 as discussed previously (Table S16), these alleles were also additive for their impacts on OY1 transcript abundance (Table S22). This observation is consistent with the hypothesis that increased OY1 abundance can overcome the negative effect of the *Oy1-N1989* allele. It is likely that multiple phenotypically-affective *cis*-acting regulatory polymorphisms at *oy1* are responsible for the *vey1* locus.

The effect sizes of the gene expression changes observed in the IBM, diverse maize inbred lines, and allele-specific expression in hybrids are quite modest, resulting in ∼10% of differences in *oy1* accumulation. If these changes in wild-type OY1 transcript accumulation are responsible for suppression of *Oy1-N1989*, then the severity of the mutant phenotype (as indicated by CCM) in the MDL F1 population should correlate with OY1 expression level. As expected, we observed a statistically significant positive correlation between the mutant derived CCM traits and OY1 counts (Table S23). Wild-type CCM in the MDL F1 population did not show any significant correlation with OY1 abundance in the emerging shoot tissue of maize inbred lines. These correlations are in agreement with the lack of any QTL at this locus controlling wild-type chlorophyll levels, and the epistatic relationship between *vey1* and *Oy1-N1989*.

## Discussion

The semi-dominant mutant allele *Oy1-N1989* encodes a dominant-negative allele at the *oy1* locus (Sawers *et al.* 2006). In a heterozygous condition, the strength of the negative effect of this allele on the MgChl enzyme complex depends on the wild-type *oy1* allele. Thus, the *Oy1-N1989* allele can sensitize maize plants to variation in MgChl and expose a phenotypic consequence for genetic variants that are otherwise invisible. Similar methodology has been adopted previously in maize to gain the genetic understanding of various traits (Chintamanani *et al.* 2010; Olukolu *et al.* 2013, 2014; Buescher *et al.* 2014). Thus, genetic screens based upon semi-dominant mutant alleles as reporters offer a cost-effective and robust approach to map QTL(s) for metabolic pathways of interest by leveraging the publicly available germplasm.

Alleles with dramatic fitness consequences may be visible to researchers working with population sizes in the thousands, such as in GWAS. By contrast, evolutionarily-relevant genetic variation may have minimal phenotypic effects in laboratory and agronomic field conditions. The cryptic variation observed by these mutant-contingent QTL approaches may not be fitness-affecting, and interpreting mutant-conditioned phenotypes as non-neutral variation would be a mistake. It is, of course, possible that neutral variants may result in increased severity of a mutant phenotype due to changes not physiologically relevant for all alleles of that reporter gene present in a species. Nevertheless, it can inform us about the allelic variation in the species and pathway topology via gene discovery.

In the current study, use of bi-parental mapping populations derived from the same inbred lines but developed using different breeding schemes provided the opportunity to compare the effect of additional rounds of random interbreeding in the development of mapping population on the genetic resolution. The comparative fine mapping of *vey1* in the Syn10 population that employed ten rounds of random mating provided far better localization of the *vey1* QTL than the IBM populations that was derived from four rounds of random mating. This observation demonstrates the benefits of increased recombination during random intermating of early generations in QTL localization. Based on these results, as future RILs are generated, we recommend increased intermating in the early generations followed by DH induction rather than relying on further recombination during the self-pollination cycles of RIL development.

This study illustrated a complementary use of line-cross QTL mapping and GWAS to explore trait genetics. As expected, GWAS provided a fine-scale genetic resolution and corroborated the mapping of *vey1*. GWAS identified two SNPs, one in the 5’ and another in the 3’ intergenic DNA, proximate to *oy1* that represent candidate quantitative trait nucleotides (QTN). The signal detection by multiple unlinked SNPs in GWAS may indicate a complex set of phenotypically-affective alleles at the *oy1* locus. Alternatively, it could be an artifact of strong associations with markers that are tightly linked to an unmeasured causative variant but unlinked or in repulsion to each other. Consistent with two QTN, rather than fortuitous linkage of tag SNPs with a single causative polymorphism, alleles at the two SNPs additively influenced chlorophyll contents in the mutant siblings. Future work will be required to identify the nucleotide changes sufficient to reproduce the *vey1* phenotype.

We used a non-destructive, inexpensive, and rapid phenotyping method to approximate leaf chlorophyll with high accuracy. Previous studies have highlighted the importance of benchmarking indirect measurements or proxies for traits of interest. For instance, near infrared reflectance spectroscopy (NIRS) that estimates major and total carotenoids in maize kernels could replace sensitive, accurate, cumbersome, expensive, slow, and destructive measurements by HPLC (Berardo *et al.* 2004). Comparisons of HPLC and NIRS measurements resulted in a correlation of 0.85 for total carotenoids (Berardo *et al.* 2004). A subjective visual score for yellow color in maize kernels was not adequate, yielding a correlation of 0.12 with HPLC measurements of total carotenoids (Harjes *et al.* 2008). Thus, using mutant alleles as reporters to develop methodologies that rely on non-invasive multispectral data as proxies for absolute quantification of biochemical compounds will accelerate studies on gene function and allele discovery. This can enable genetic studies that are currently deemed unfeasible due to the arduous task of phenotyping large populations for traits only visible in the laboratory.

### How could a 10% change in wild-type OY1 expression affect chlorophyll biosynthesis in the Oy1-N1989/oy1 mutant heterozygotes?

Magnesium chelatase (MgChl) is formed by a trimer of dimers of MgChl subunit I interacting with the other subunits of MgChl complex. A previous study found that addition of mutant or wild-type BCHI protein to pre-assembled MgChl complexes resulted in altered reaction rates due to differences in subunit turnover, which occurred on a minutes time-scale (Lundqvist *et al.* 2013). This subunit turnover and reformation of the complex dynamically exchange mutant and wild-type BCHI subunits over time. Therefore, any net increase in the amount of wild-type OY1 in the reaction pool, for instance, due to higher transcription of wild-type *oy1* allele will allow a higher rate of magnesium chelatase activity and result in more chlorophyll biosynthesis. The observation of stronger affinity and greater dissociation rate of BCHI${}^{L111F}$ subunits (orthologous to the L176F change encoded by *Oy1-N1989*) for the wild-type subunits (Hansson *et al.* 2002) suggests that exchange of BCHI monomeric units in the magnesium chelatase complex might also differ based on the structure of BCHI protein (Lundqvist *et al.* 2013). In the AAA+ protein family, the ATP-binding site is located at the interface of two neighboring subunits in the oligomeric complex (Vale 2000). Since a dimer of functional MgChl subunit I proteins are required for the complex to carry out the MgChl activity (Lundqvist *et al.* 2013), approximately 1 in 3 dimers of assembled MgChl subunit I will be active in a 1:1 mixture of wild-type and BCHI${}^{L111F}.$ Indeed, complexes made from reaction mixtures with equal proportions of wild-type and BCHI${}^{L111F}$ subunits resulted in ∼26% of the enzyme activity of an equivalent all-wildtype mix (Lundqvist *et al.* 2013). Therefore, we expect that decreasing expression of the wild-type *oy1* subunit by 10% and creating a 0.9:1.1 mixture of wild-type OY1 and mutant OY1-N1989 protein, respectively, would result in ∼21% activity compared to the activity of all-wildtype mixture. Likewise, increasing the wild-type *oy1* expression by 10% would result in ∼30% activity of MgChl compared to the all-wildtype mixture. This dosage-sensitivity is a general feature of protein complexes (Birchler and Newton 1981; Grossniklaus *et al.* 1996; Veitia 2003; Birchler and Veitia 2012), and the semi-dominant nature of *Oy1-N1989* is dosage sensitive. Taking these observations and proposed models on the dynamics of molecular interaction between the wild-type and mutant BCHI protein subunits (especially BCHI${}^{L111F}$) into account, it is formally possible that a small change in the expression of wild-type OY1 can have a significant impact on the magnesium chelatase activity of heterozygous *Oy1-N1989/oy1* plants. The increase in magnesium chelatase activity due to the even small relative increase in the proportion of wild-type OY1 transcripts over the mutant OY1-N1989 transcripts will read out as a proportional, presumably non-linear, increase in chlorophyll accumulation.

### What is vey1?

Variation at the *vey1* locus appears to be the result of allelic diversity linked to the *oy1* locus. The only remaining possibilities are regulatory polymorphisms within the *cis*-acting control regions of *oy1*. Previous studies have utilized reciprocal test-crosses to loss-of-function alleles in multiple genetic backgrounds to provide single-locus tests of additive QTL alleles in an otherwise identical hybrid background (Dilkes *et al.* 2008). Protein-null alleles of *oy1* isolated directly from the B73 and Mo17 backgrounds could be used to carry out a similar test. The intergenic genomic region in maize is spanned by transposable elements and can be highly divergent between different inbred lines due to large insertion/deletion polymorphisms (SanMiguel and Bennetzen 1998). Consistent with this, inbreds B73 and Mo17 are polymorphic at the region between *oy1* and *gfa2*. These two maize inbred lines share ∼12 kb of sequence interspersed with numerous large insertions and deletions that add ∼139 kb of DNA sequence to Mo17 as compared to B73 (data not shown). However, we did not find any conserved non-coding sequence (CNS) in this region (data not shown). It is conceivable that recombinants at *oy1* between B73 and Mo17 themselves could be identified and used to test the effects of upstream or downstream regulatory sequences. The recombinant haplotypes encoding all four possible alleles at the top two SNPs identified in the GWAS indicate that Mo17 may be a strong enhancer due to more than one causative polymorphism. Allele-specific expression at *oy1* locus in the *Oy1-N1989* heterozygous mutants demonstrated the existence of functional *cis*-acting regulatory polymorphism between *Oy1-N1989* and both wild-type *oy1* alleles in B73 and Mo17. In addition, *oy1* is affected by a *cis*-eQTL in the IBM and MDL. Together with our other data, the allele-specific expression at OY1 that was visible when we re-analyzed the data from Waters *et al.* (2017) led us to propose that *vey1* encodes a *cis*-acting regulatory DNA sequence variation (Figure S9). Sequence comparisons outside the protein coding sequence of the gene can be quite challenging, especially in maize, as it exhibits limited to poor sequence conservation between different inbred lines (SanMiguel *et al.* 1996). Thus, distinguishing phenotypically-affective polymorphisms from the neutral variants, *e.g.*, via transgenic testing, is not trivial. As a result, biochemical and *in vitro* studies are the best tool for functional validation of these polymorphisms (Wray *et al.* 2003). Similar experiments have been done to characterize the role of DNA sequence polymorphisms *in cis* on the expression of downstream genes in the case of *flowering locus T* in *Arabidopsis* and *teosinte branched1* in maize (Adrian *et al.* 2010; Studer *et al.* 2011).

### Relevance to research on transcriptional regulation

Since their discovery, the role of regulatory elements in gene function has been recognized as vital to our understanding of biological systems (McClintock 1950, 1956a,b, 1961; Peterson 1953; Jacob and Monod 1961). Gene regulation and gene product dosage are at the forefront of evolutionary theories about sources of novelty and diversification (Ohno 1972; King and Wilson 1975). Transcriptional regulation of a gene can be as important as the protein coding sequence (Wray *et al.* 2003). For instance, complete knock-down of expression of a gene by a regulatory polymorphism will have the same phenotypic consequence as the non-sense mediated decay of a transcript harboring an early stop codon (Willing *et al.* 1996). We do not have a set of rules, analogous to codon tables, for functional polymorphisms outside the coding sequence of a gene. Thus, detecting expression variation and tying it to phenotypic consequence, especially in the absence of CNS, remains a challenge to this day.

Several eQTL studies in eukaryotes have found abundant cis and *trans*-acting genomic regions that affect gene expression (Brem *et al.* 2002; West *et al.* 2007; Li *et al.* 2013, 2018). Expression polymorphisms are a potential source of phenotypic variation (Gibson and Weir 2005), and multiple studies detected expression polymorphisms co-segregating with phenotypic changes, including contributions to species domestication (Clark *et al.* 2006; Salvi *et al.* 2007; Schwartz *et al.* 2009; Lemmon *et al.* 2014). However, the majority of the *cis*-eQTLs only exhibit a moderate difference in gene expression. Detecting such variants and linking them to visible phenotypes may require detailed study using approaches focused on the specific biological process affected by the gene product. We do not yet have a standardized experimental tool for these purposes. As such, we cannot simultaneously identify and characterize the phenotypic impact of most *cis*-acting eQTLs. The *vey1* polymorphism detected in the current study co-segregates with a *cis*-eQTL at *oy1* in IBM (Li *et al.* 2013, 2018) and diverse maize inbred lines (Kremling *et al.* 2018). The high heritability of the alternative and direct CCM phenotype allowed us to scan large populations at a rapid rate to identify the genomic regions underlying the *cis*-acting regulatory elements and study allelic diversity in the natural population of maize. We propose that the approach we have taken is not likely to be unique to *oy1*. Therefore, we propose that all semi-dominant mutant alleles can be used as reporters to not only detect novel *cis*-acting gene regulatory elements but also functionally validate previously-detected *cis*-eQTL(s) from genome-wide eQTL studies.
